# T Lymphocyte Antigen 4-Modified Dendritic Cell Therapy for Asthmatic Mice Guided by the CCR7 Chemokine Receptor

**DOI:** 10.3390/ijms150915304

**Published:** 2014-08-29

**Authors:** Yan Chen, Yongming Wang, Zhou Fu

**Affiliations:** 1 Department of Respiratory Medicine, Children’s Hospital of Fudan University, Shanghai 201102, China; E-Mail: yanchenscience@hotmail.com; 2 Department of Neonatology, Children’s Hospital, Chongqing Medical University, Chongqing 404100, China; E-Mail: yongmingwangscience@hotmail.com; 3 Department of Respiratory Medicine, Children’s Hospital, Chongqing Medical University, Chongqing 404100, China; 4 Respiratory Research Laboratory, Ministry of Education Key Laboratory of Child Development and Disorders, Chongqing 404100, China

**Keywords:** the cytotoxic T lymphocyte antigen 4 (CTLA4), asthma, chemokine receptor CCR7, dendritic cells (DCs), recombinant viral vectors, precision-guided immunotherapy

## Abstract

The CD80/CD86-CD28 axis is a critical pathway for immuno-corrective therapy, and the cytotoxic T lymphocyte antigen 4 (CTLA4) is a promising immunosuppressor targeting the CD80/CD86-CD28 axis; however, its use for asthma therapy needs further optimization. A human CTLA4 fused with the IgCγ Fc (CTLA4Ig) and mouse CC chemokine receptor type7 (CCR7) coding sequences were inserted into a recombinant adenovirus (rAdV) vector to generate rAdV-CTLA4Ig and rAdV-CCR7. The naive dendritic cells (DCs) were infected with these rAdVs to ensure CCR7 and CTLA4Ig expression. The therapeutic effects of modified DCs were evaluated. rAdV-CTLA4Ig and rAdV-CCR7 infected DCs improved all asthma symptoms. Inflammatory cell infiltration and cytokine analysis showed that rAdV-CTLA4Ig and rAdV-CCR7-modified DC therapy reduced the number of eosinophils and lymphocyte and neutrophil infiltration in the lung. Interestingly, assessment of the humoral immunity showed that the IL-4 and IFNγ levels of the rAdV-CTLA4Ig and rAdV-CCR7-modified DC-treated mice decreased significantly and did not reverse the Th1/Th2 balance. DCs expressing CCR7 displayed guidance ability for DC migration, primarily for DCs in the inflammatory lung. Additionally, the rAdVs caused an inflammatory response by inducing DC differentiation, inflammatory cell infiltration and changes in cytokines; however, mice transplanted with rAdV-green fluorescent protein (GFP)-infected DCs displayed no asthma manifestations. In conclusion, CTLA4Ig-modified DCs exhibited a therapeutic effect on asthma, and CCR7 may guide DC homing. The combination of these two molecules may be a model for precision-guided immunotherapy.

## 1. Introduction

Human allergic asthma is characterized by airway inflammation, airway hyperresponsiveness and reversible airway intermittent obstruction [1,2,3]. Chronic allergic asthma may lead to airway remodeling, including goblet cell hyperplasia, airway wall fibrosis, smooth muscle thickening and vascular proliferation [3,4]. Allergic asthma seriously affects the life quality of patients and also causes a variety of complications, such as pulmonary emphysema, chronic pulmonary heart disease and respiratory failure [5,6]. Currently, the clinical treatment of asthma is limited [7].

Although the cellular and biochemical processes underlying chronic inflammation and airway remodeling are poorly understood, recent studies suggest that cellular immunity participates in the pathophysiologic process of asthma. Animal experiments indicate that allergic asthma is related to an inappropriate balance between the allergen-mediated activation of T helper type 1 (Th1) and type 2 (Th2) cells and reduced or poorly functioning Th2/regulatory T cells [8,9]. Th17 cells promote neutrophilic inflammation in concert with Th2 cells which are important in the development of airway hyper-responsiveness [10]. Allergic sensitization through the airway promotes Th17 response and IL-17F-deficient mice have an impaired neutrophilic response to allergen [10]. Human studies have shown increased expression of IL-17A and IL-17F in bronchial submucosa in moderate to severe asthma [11,12]. Increased airway hyperreactivity in response to methacholine in patients with asthma positively correlates with IL-17A levels in the sputum [13] and a polymorphism in IL-17F that results in a loss-of-function mutation is inversely related to asthma risk [14]. Furthermore, recent evidence suggests that the type-2 immune response is initiated by epithelial cell-derived cytokines such as IL-25, IL-33 and thymic stromal lymphopoietin [15].

The development and activation of T cells require an ordered series of signals from antigen-presenting cells (APCs), such as dendritic cells (DCs) [16,17]. These are summarized as follows: a required but insufficient signal, T-cell receptor (TCR) binding to a specific antigen displayed on the antigen major histocompatibility complex (MHC) delivered by DCs, and essential signals delivered by co-signaling molecules. These cell-surface glycoprotein molecules can direct, modulate and fine-tune TCR signals [18,19,20]. Based on their functional outcome, co-signaling molecules can be divided into co-stimulators, which result in clonal expansion, augmented cytokine secretion, and enhanced cell survival of activated T cells, and co-inhibitors, which suppress T-cell activation [12]. Therefore, DC co-signaling molecules control the priming, differentiation, and growth and functional maturation of the T cell response spatio-temporally, and they are also a possible target for immuno-corrective therapy [21,22].

The CD80/CD86-CD28 axis is a critical target for immuno-corrective therapy, because CD28 engaged by CD80 or CD86 concomitant with TCR signaling is sufficient to fully activate resting naive T cells. Inhibition of the CD80/CD86-CD28 axis prevents T-cell activation *in vitro* and *in vivo* [18,21,22]. Studies have shown that CD28 is essential for the development of allergic airway inflammation in a number of preclinical models [23,24]. The CTLA4, an anti-CD28 antagonist, had one log higher competitive binding activity to CD80/CD86 than CD28 and is widely used in immuno-corrective therapy [18]. Numerous studies demonstrated that treatment with various species of CTLA4Ig, a soluble CTLA4 immunoglobulin fusion protein molecule, had effects in several diseases, such as preventing contact hypersensitivity, acquired immune deficiency syndrome, psoriasis vulgaris and asthma [25,26,27,28].

Although immuno-corrective therapy based on the CD80/CD86-CD28 axis has shown encouraging results both in clinical and experimental studies, the administration strategy must be further improved. The positioning of DCs and differentiated T cells within tissues is important for the efficiency of the adaptive immune responses [18,19,20,21,22]. The CC chemokine receptor type 7 (CCR7) is essential for the homing of antigen-experienced T cells to lymphoid and non-lymphoid destinations, and it also contributes to the precise functioning of the adaptive immune responses [29,30,31]. The expression characteristics and the role of CCR7 in DCs are poorly understood, and a limited number of studies showed that after blockade of the CD80/CD86-CD28 axis, CCR7 expression is decreased [32], to optimize CD80/CD86-CD28 axis based immuno-corrective therapy, in this study, we generated a recombinant adenovirus vector harboring the human CTLA4Ig chimeric DNA expression fragment. Additional adenovirus vector harboring the mouse CCR7 coding sequence was also constructed. After modification of the DCs using these viral vectors, the therapeutic effects of CCR7-guided CTLA4Ig were evaluated in a mouse asthma model.

## 2. Results

### 2.1. Quality Control of Recombinant Adenoviruses (rAdVs)

**Figure 1:**
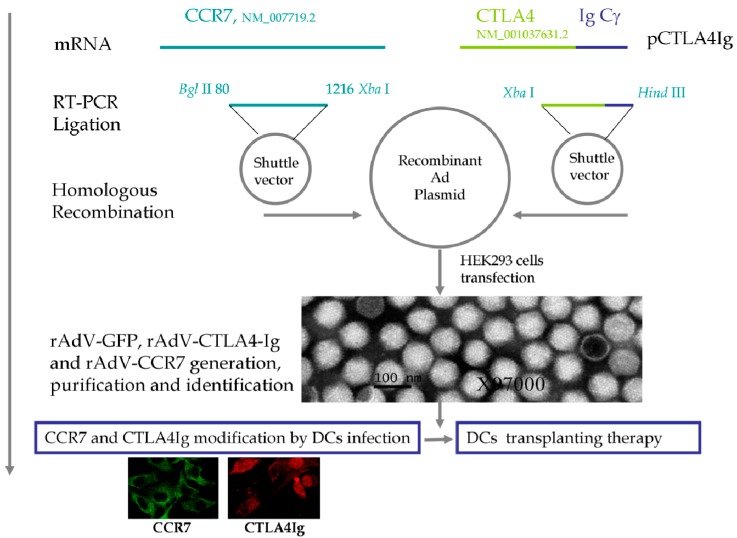
Schematic diagram of the vectors and therapeutic strategy. The ecto-domain of the human CTLA4 gene (NM_001037631.2) was fused with the coding sequence of IgCγ Fc, The full-length mouse CCR7-coding sequence (NM_007719.2) was cloned. The two DNA fragments were inserted into the shuttle vector and then transferred to the recombinant adenovirus plasmid by homologous recombination. rAdV-CCR7, rAdV-CTLA4, and the control rAdV-GFP were produced and purified. After verification of the inserts and virus, dendritic cells (DCs) were infected with these viruses to upregulate the expression of CCR7 and CTLA4Ig.

Purified rAdVs were verified by transmission electron microscopy, as showed in Figure 1. The rAdVs displayed typical topological characteristics of adenoviruses, with a diameter of 80 nm. After rAdV genomic DNA extraction, the inserts, CCR7 or CTLA4Ig, were removed using Bgl II and Xba I or Xba I and Hind III. The DNA fragments were confirmed by DNA sequencing. To determine the optimal MOI, rAdV-GPF, rAdV-CCR7 and rAdV-CTLA4Ig were titrated by plaque forming assay. The expression of GFP, CCR7, and CTLA4Ig from the rAdVs were verified in HEK293 cells. All rAdVs showed high transduction efficacy and high stable expression of the targeting proteins at an MOI 100:1 as reported previously [8].

### 2.2. Therapeutic Dendritic Cell (DC) Modification

For DC induction, each cell culture well was seeded with 3 × 10^5^ immature DCs. After 7 days of stimulation with 20 ng/mL rmGM-CSF and 10 ng/mL rmIL-4, the DCs were subjected to fluorescence-activated cell sorting (FACS) analysis. Over 90% of the DCs were CD11c positive. For DC modification, the above DCs were infected with an MOI of 100 of rAdV-CCR7 and rAdV-CTLA4Ig. After 2 days of incubation, the CCR7 and CTLA4Ig expression on the DC surface and cytoplasm was ascertained by FACS and immune cellular histochemical analysis. For the DC surface, 10.86% ± 1.03% and 56.99% ± 1.42% of the DCs infected with rAdV-CTLA4Ig and rAdV-CCR7 expressed CTLA4Ig and CCR7, respectively, whereas for the ovalbumin- (OVA-) and rAdV-GFP-infected DCs, only 10.19% ± 0.41% to 11.82% ± 0.73% expressed CCR7, respectively, and neither expressed CTLA4 (Table 1). In the DC cytoplasm, 80.00% ± 5.34% and 90% ± 4.52% of the DCs infected with rAdV-CTLA4Ig and rAdV-CCR7 expressed CTLA4Ig and CCR7, respectively, whereas for rAdV-GFP-infected DCs, neither CTLA4 nor CCR7 was detected (Table 1 and Figure 1). In summary, DCs expressed undetectable CTLA4 and less CCR7 without rAdV-CTLA4Ig and rAdV-CCR7 modification. Together with our previous report [8], the CTLA4Ig was secreted from the DCs and was maintained at 40 to 80 ng/mL in the supernatant. The high level of plasma CTLA4Ig may be storage for secretion, and only very low levels of CTLA4Ig were detected on the DC surface.

**Table 1:** The expression characteristics of cytotoxic T lymphocyte antigen 4-immunoglobulin (CTLA4Ig) and CC chemokine receptor type 7 (CCR7) in dendritic cells (DCs).

Cellular Localization	CTLA4Ig (%)	CCR7 (%)	Both CTLA4Ig and CCR7 (%)
DC surface			
rAdV-CTLA4Ig and rAdV-CCR7	10.86 ± 1.03	56.99 ± 1.42	10.04 ± 0.32
rAdV-GFP	0.00 ± 0.01	11.82 ± 0.73	0.00 ± 0.00
OVA	0.00 ± 0.00	10.19 ± 0.41	0.00 ± 0.01
DC cytoplasm			
rAdV-CTLA4Ig and rAdV-CCR7	80.00 ± 5.34	90.00 ± 4.52	75.00 ± 5.12
rAdV-GFP	0.00 ± 0.01	0.00 ± 0.01	0.00 ± 0.00

DCs, dendritic cells; OVA, ovalbumin; rAdV, recombinant adenovirus; GFP, green fluorescent protein; CTLA4Ig, cytotoxic T lymphocyte antigen 4-immunoglobulin; CCR7, CC chemokine receptor type 7. All measurements were repeated at least 3 times.

### 2.3. Surface Expression of DC Activation Markers after in Vitro Modification

After 7 days of incubation with 20 ng/mL recombinant murine granulocyte-macrophage colony-stimulating factor (rmGM-CSF) and 10 ng/mL rmIL-4, the positive rates for CD40, CD80, CD86 and MHC of DCs were 3.27% ± 0.21%, 63.74% ± 2.35%, 19.89% ± 1.10%, and 52.50% ± 2.32%, respectively, which showed the characteristic of DCs (Table 2). After 2 additional days of incubation with OVA, rAdV-GFP, rAdV-CCR7 and rAdV-TCLA4Ig, the CD proteins expression pattern was determined by FACS analysis. The expression of CD40, CD80, CD86 and major histocompatibility complex (MHC) on DCs infected with rAdV-CCR7 and rAdV-TCLA4Ig increased to 19.59% ± 1.52%, 66.51% ± 2.21%, 36.90% ± 3.01%, and 96.69% ± 6.01%, respectively (Table 2). To compare the DCs differentiation, DCs incubated with OVA and control rAdV-GFP were also subjected to FACS analysis, the positive rates for CD40, CD80, CD86 and MHC were 4.43% ± 0.11% and 51.98% ± 4.55%, 93.52% ± 5.22% and 96.41% ± 5.01%, 30.87% ± 1.01% and 95.08% ± 4.66%, and 85.40% ± 2.33% and 98.56% ± 6.12%, respectively (Table 2). In summary, ovalbumin (OVA), recombinant adenovirus-green fluorescent protein (rAdV-GFP) and recombinant adenovirus-cytotoxic T lymphocyte antigen 4-immunoglobulin (rAd-CTLA4Ig) and recombinant adenovirus-CC chemokine receptor type 7 rAdV-CCR7 displayed different induction characteristics for DCs. OVA increased the expression of CD80 and MHC, whereas rAdV-GFP promotes CD40, CD80, CD86 and MHC expression. CD40 and CD86 expression in AdCTLA4Ig- and AdCCR7-infected DCs is higher than that in OVA-treated DCs and lower than rAdV-GFP-infected DCs. CD80 expression in AdCTLA4Ig- and AdCCR7-infected DCs is lower than that in OVA- and rAdV-GFP-infected DCs.

### 2.4. Mouse Asthma Model and Modified DC Therapy

**Figure 2:**
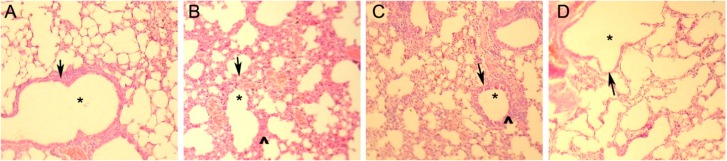
H&E staining. Bloodshot, inflammatory cell infiltrate and bronchial tube pulmonary alveolus structural changes were ascertained by histopathology, asterisk indicates the bronchial tube, arrows indicates the bronchial wall, ^ indicates interstitial edema and inflammatory cell infiltration. (**A**) healthy control mice; (**B**) asthmatic mice; (**C**) rAdV-GFP infected DC therapy mice; and (**D**) rAdV-CCR7 and rAdV-CTLA4Ig infected DC therapy mice.

The mice asthma model was generated by OVA sensitization as described previously [8]. OVA-sensitized mice showed dysphoria or asthenia, nodding with breathing and forelimb shrinkage manifestations when re-challenged with OVA (Table 3). The mice transplanted with rAdV-GFP-infected DCs displayed mild asthma manifestations and slight pathological damage to the lung, although the *in vitro* experiments described above showed that rAdV infection robustly activated DCs (Table 3). For the mice sensitized with OVA and transplanted with rAdV-CCR7- and rAdV-TCLA4Ig-infected DCs before OVA challenge, all asthma symptoms, including dysphoria or asthenia, nodding with breathing, forelimb shrinkage and gatism were improved greatly, and the respiratory rate decreased to 120 ± 19 beats per minute, which is similar to the normal control (Table 3). The lungs of mice sensitized and challenged with OVA appeared bulging and bloodshot with bronchial tube pulmonary alveolus structural damage (Figure 2). Of the mice sensitized with OVA and transplanted with rAdV-CCR7- and rAdV-TCLA4Ig-infected DCs before OVA challenge, the pathological damage in the lung was significantly less (Figure 2). In summary, the transfusion of rAdV-CCR7- and rAdV-TCLA4Ig-infected DCs displayed a therapeutic effect for asthma.

**Table 2:** Surface expression of DC activation markers.

DC Activation Markers	GM-CSF and IL-4	OVA	rAdV-GFP	rAdV-CTLA4Ig and rAdV-CCR7
**Induction duration (days)**	7	2	2	2
**CD40 (%)**	3.27 ± 0.21	4.43 ± 0.11	51.98 ± 4.55	19.59 ± 1.52 *^,#^
**CD80 (%)**	63.74 ± 2.35	93.52 ± 5.22	96.41 ± 5.01	66.51 ± 2.21 *^,#^
**CD86 (%)**	19.89 ± 1.10	30.87 ± 1.01	95.08 ± 4.66	36.90 ± 3.01 *
**MHC II (%)**	52.50 ± 2.32	85.40 ± 2.33	98.56 ± 6.12	96.69 ± 6.01

Final concentrations for GM-CSF, IL-4 and OVA were 30 ng/mL, 15 ng/mL and 1 μg/mL, respectively. DC, dendritic cells; CD, cluster of differentiation; GM-CSF, granulocyte-macrophage colony-stimulating factor; IL, interleukin; OVA, ovalbumin; Ad, adenovirus; GFP, green fluorescent protein; CTLA4Ig, cytotoxic T lymphocyte antigen 4-immunoglobulin; CCR7, CC chemokine receptor type 7; MHC II, major histocompatibility complex class II molecules. All measurements were repeated at least 3 times. *, *p* < 0.05, compared with rAdV-GFP group; ^#^, *p* < 0.05, compared with OVA group.

**Table 3:** Symptoms and pathology observation.

Symptoms and Pathology	Control	Asthma	rAdV-GFP	rAdV-CTLA4Ig and rAdV-CCR7
	(*n* = 8)	(*n* = 8)	(*n* = 8)	(*n* = 8)
Asthma manifestations				
Dysphoria or asthenia	No	Yes	Yes	improved
Nodding with breathing	No	Yes	No	improved
Camponotus erect	No	Yes	No	improved
Forelimb shrinkage	No	Yes	No	improved
Gatism	No	Yes	No	improved
Respiratory rate (beats per minute)	115 ± 13	180 ± 25	121 ± 16	120 ± 19
Pathological damage of lung				
Bulging and bloodshot	No	Yes	No	improved
Inflammatory cell infiltration	No	Yes	Yes	improved
Bronchial tube pulmonary alveolus structure	Nomal	Pathological changes	Normal	Lesion improvement

IL, interleukin; IFN, interferon; CTLA4Ig, cytotoxic T lymphocyte antigen 4-immunoglobulin; CCR7, CC chemokine receptor type 7; GFP, green fluorescent protein.

### 2.5. Cell Composition and Cytokine Changes

To observe the cellular and humoral immunity changes in bronchoalveolar lavage fluid (BALF) and sera of all mouse groups, both the BALF and sera of the mice were collected and subjected to cell composition and cytokine analysis. For the BALF, the total white blood cells, eosinophils, lymphocytes and neutrophils of the asthma group were 155.93 ± 5.80, 17.05 ± 1.59, 92.25 ± 6.90 and (46.63 ± 7.65) × 10^4^ per·mL, respectively, which were significantly higher than the control mice (*p* < 0.01). The IL-4 and IFNγ levels of the asthma group were significantly higher and lower than that of control mouse group, respectively (*p* < 0.01) (Table 4). The above indices of the rAdV-GFP-infected DC-treated mouse group are similar to the asthma mouse group, suggesting that rAdV-GFP causes cellular and humoral immunity changes (Table 4). The total white blood cells, eosinophils, lymphocyte and neutrophil of the of rAdV-CCR7 and rAdV-CTLA4Ig infected DC treated mice were 18.89 ± 2.17, 0.50 ± 0.24, 8.11 ± 1.12 and (10.28 ± 2.62) × 10^4^ per·mL, respectively, which was similar to the normal control and significantly lower than the asthma and rAdV-GFP-infected DC-treated mouse groups (*p* < 0.01). The IL-4 level was 84.02 ± 17.30 pg/mL, which was similar to the control group, and the IFNγ level was 55.87 ± 12.8 pg/mL, which was significantly lower than all the other groups (*p* < 0.01) (Table 4). There is no significant difference in the cellular composition and cytokine levels for the sera of all the groups (Table 4).

**Table 4:** Cell classification and cytokine level of mice groups.

Cell Classification and Cytokine Level	Control	Asthma	rAdV-GFP	rAdV-CTLA4Ig and rAdV-CCR7
	(*n* = 10)	(*n* = 10)	(*n* = 10)	(*n* = 10)
BALF				
Total white blood cells (×10^4^/mL)	21.8 ± 3.25	155.93 ± 5.80	165.49 ± 7.00	18.89 ± 2.17 *^,#^
Eosinophils (×10^4^ mL)	0.45 ± 0.23	17.05 ± 1.59	20.74 ± 3.53	0.50 ± 0.24 *^,#^
Lymphocyte (×10^4^ mL)	10.94 ± 2.94	92.25 ± 6.90	91.13 ± 6.81	8.11 ± 1.12 *^,#^
Neutrophil (×10^4^ mL)	10.41 ± 2.47	46.63 ± 7.65	53.63 ± 5.21	10.28 ± 2.62 *^,#^
BALF				
IL-4 (pg/mL)	93.28 ± 14.01	156.08 ± 51.78	161.18 ± 56.68	84.02 ± 17.30 *^,#^
IFNγ (pg/mL)	162.16 ± 62.14	104.25 ± 14.99	98.12 ± 14.74	55.87 ± 12.8 *^,#^
Sera				
IL-4 (pg/mL)	36.47 ± 1.46	39.82 ± 1.43	36.19 ± 2.94	35.92 ± 1.93
IFNγ (pg/mL)	65.27 ± 3.31	52.75 ± 1.93	48.78 ± 4.17	55.27 ± 3.31

BALF, bronchoalveolar lavage fluid; IL, interleukin; IFN, interferon; CTLA4Ig, cytotoxic T lymphocyte antigen 4-immunoglobulin; CCR7, CC chemokine receptor type 7; GFP, green fluorescent protein. *, *p* < 0.01, compared with rAdV-GFP group; and ^#^, *p* < 0.01, compared with OVA group.

### 2.6. CCR7 Guides DC Migration

**Figure 3:**
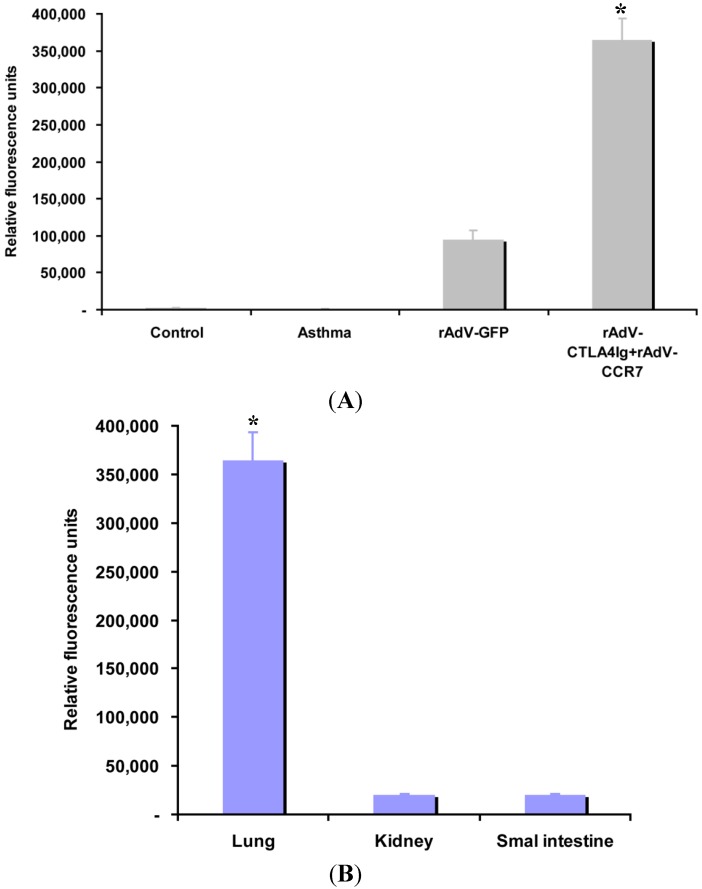
CCR7 guides DC migration, *, *p* < 0.05 *versus* other groups or organs. (**A**) The relative mean fluorescence intensity in the lungs of the control, asthma, rAdV-GFP-, rAdV-CCR7- and rAdV-CTLA4Ig-infected DC-treated mice; and (**B**) lung specific DC migration.

To evaluate whether CCR7 could guide DCs migration to the inflammatory lung, the transplanted DCs distribution was assessed by GFP tracking. The relative mean fluorescence intensity of the control, asthma, rAdV-GFP-, rAdV-CCR7- and rAdV-CTLA4Ig-infected DC-treated mice are 2070 ± 141, 1131 ± 125, 94,975 ± 12,006 and 364,563 ± 29,302 (relative fluorescence units, RFU), respectively (Figure 3A). rAdV-CCR7- and rAdV-CTLA4Ig-infected DCs are highly concentrated in the lung compared to rAdV-GFP (*p* < 0.05). To further assess the distribution of rAdV-CCR7 infected DCs, the mean fluorescence intensity of GFP in multiple organs was evaluated. The relative mean fluorescence intensity of the lung, kidney and small intestine are 364,563 ± 29,302, 19,857 ± 1341 and 20,550 ± 1150 RFU, respectively (Figure 3B), which suggests that DCs are primarily distributed in the lung. In summary, these data suggest that CCR7 guides CCR7-modified DCs to the lung.

## 3. Discussion

Inappropriate humoral and cellular immunity are induced by many allergens and play a role in the gradual deterioration in many chronic allergic diseases [33,34]. Currently, molecular and cellular immuno-corrective therapeutic approaches are under development. Intravenous administration of CTLA4Ig showed significant effects in patients with psoriasis vulgaris [27], and animal experiments demonstrated that intravenous infusion of CTLA4Ig reverses asthma manifestations and inhibits airway eosinophilia and hyperresponsiveness by regulating the development of Th1/Th2 subsets [8]. DCs are versatile controllers of the innate and adaptive immune responses and have emerged as the key cell type in adaptive cellular immunotherapy [35,36]. Genetically engineered CTLA4 DCs were utilized in many studies, including asthma therapy [8]. CTLA4Ig-modified DCs attenuated allergic airway inflammation and hyperresponsiveness in a murine model of asthma [8,9].

In this study, transplantation of rAdV-CTLA4Ig and rAdV-CCR7-modified DCs significantly improved the pathological damage of lung and asthma manifestations. Inflammatory cell infiltration analysis showed that rAdV-CTLA4Ig and rAdV-CCR7-modified DCs therapy decreased the inflammatory response by reducing the number of eosinophils and lymphocyte and neutrophil infiltration. *In vitro* data showed that rAdV-CTLA4Ig and rAdV-CCR7-modified DCs secrete CTLA4Ig which blocks CD86 expression without influencing the expression of MHC-II on the surface of DCs, which is similar to the results of the DCs modified by CTLA4Ig alone in our previous study [8]. *In vivo* data showed that, the level of IL-4, a Th2 cytokine, in rAdV-CTLA4Ig and rAdV-CCR7-modified DC-treated mice decreased significantly compared to that of asthmatic mice, which is also consistent with our previous report that the DCs modified by rAdV-CTLA4Ig alone could also suppress IL-4 increasing in asthma mouse [8]. Interestingly, the level of IFNγ, a Th1 cytokine, in the BALF of mouse treated with DCs modified by rAdV-CTLA4Ig alone was significantly higher than that of asthma mouse in our previous study [8]. In this study, the IFNγ level in rAdV-CTLA4Ig and rAdV-CCR7-modified DC-treated mice was significantly lower than that of asthma, control or rAdV-GFP treated mouse. The Th1/Th2 ratios of the control, asthma, rAdV-GFP and rAdV-CTLA4Ig and rAdV-CCR7 mouse were 1.74, 0.67, 0.61 and 0.66, respectively. Thus, rAdV-CTLA4Ig and rAdV-CCR7-modified DC-treatment improvment in the pathophysiologic process of asthma was not via reversing the Th1/Th2 balance. The above phenomenon may be caused by CCR7 incorporation. Alternatively, there are studies that challenge the Th1/Th2 imbalance theory [37,38,39].

CCR7 may guide T cell exit from peripheral tissues and entry into afferent lymphatics [29,30,31]. Studies suggest that lamina propria DCs with unique immunomodulatory activities migrate to mesenteric lymph nodes in a CCR7-dependent manner to engage in the presentation of intestinal epithelial cell associated antigens acquired in the lamina propria [29]. In our study, CCR7-upregulated DCs displayed guidance for DC migration, primarily for DCs in the inflammatory site, *i.e.*, the lung, which suggests that CCR7 may be a good accessory molecule for targeting immuno-corrective therapy.

*In vitro* DC induction data suggest that OVA stimulation induces CD80 expression predominantly, whereas rAdV-CTLA4Ig- and rAdV-CCR7-infected DCs displayed a slight increase in CD80 and CD86 expression. By contrast, rAdV-GFP-infected DCs upregulated the expression of CD40, CD80, CD86 and MHC. Together with the inflammatory cell infiltration and cytokine analysis data, rAdV infection or rAdV-modified DC infusion may lead to inflammatory reaction, whereas DCs expressing CCR7 and CTLA4Ig can offset the inflammatory response caused by rAdV infection and improve the physiopathological injury of asthma.

Dysregulated T helper type 2 (Th2)-biased immune responses directed against antigens and impaired CD4^+^CD25^+^ forkhead box P3 (FoxP3)^+^ Treg cells play important roles in the development of asthma [8,40]. In this study, our primary objective was to determine whether CCR7 contributes to DC homing; however, we have not performed an analysis of Treg cell differentiation. Moreover, measurement of airway hyperresponsiveness and mixed lymphocyte reaction assessments are planned for our future studies.

A recent randomized controlled trial suggested that intravenous infusion of CTLA4Ig had no effect on the clinical measures or severity of asthma symptoms [41]. This conflicting report may be because of the differences between humans and mice, the CTLA4 administration route and strategy and other unknown factors. In human asthma, the CD80/CD86-CD28 axis blockade via intravenous infusion CTLA4Ig may be counteracted by an integral immune compensatory mechanism. By contrast, secondary reaction after intravenous infusion CTLA4Ig remains unknown because it blocks the CD80/CD86-CD28 axis.

## 4. Experimental Section

### 4.1. Mice

Six- to eight-week-old female BALB/c (H-2Kd, I-Ad) mice weighing 17.4 ± 0.6 g were maintained under specific-pathogen-free (SPF) conditions at the Chongqing Medical University Animal Resources Centre and raised on an OVA-free diet (Sigma, St Louis, MO, USA). All animal experiments were conducted in accordance with internationally recognized guidelines for animal experiments (“Animal Research: Reporting *in Vivo* Experiments” (ARRIVE) guidelines) and were approved by the Animal Ethics Committee of Chongqing Medical University (reference number 2008-0016).

### 4.2. Recombinant CTLA4Ig and CCR7 Adenovirus Vectors

The ecto-domain of the human CTLA4 gene (NCBI Reference Sequence: NM_001037631.2) was fused to the coding sequence of IgCγ Fc to ensure CTLA4Ig secretion. This DNA fragment was inserted into a replication-defective recombinant adenovirus (Agilent Technologies, La Jolla, CA, USA) to generate the CTLA4Ig viral vector rAdV-CTLA4Ig. The full length mouse CCR7 coding sequence (NCBI Reference Sequence: NM_007719.2) was cloned by reverse transcription PCR using the following primers: F, 5'-AGATCTATGGACCCAGGGAAACCCAGGAAAAAC-3' and R, 5'-TCTAGACTACGGGGAGAAGGTTGTGGTGGTC-3' with Bgl II and Xba I sites in each primer (synthesized by Biovisualab, Shanghai, China). After sequence confirmation, the DNA fragment was inserted into a replication-defective recombinant adenovirus to generate the CCR7 viral vector rAdV-CCR7. For all rAdVs, a *green fluorescent protein* (*GFP*) gene was inserted by Agilent Technologies to monitor the infection efficiency of the recombinant viruses. The empty rAdV with GFP (rAdV-GFP) was used as a control for the influence of viral infection on immuno-corrective therapy. All viral vectors were transfected into HEK293 cells and amplified three to five times in the same cell line. After purification, the rAdVs were tittered by plaque-forming assay and stored at −80 °C until use.

### 4.3. DC Isolation, Induction and Modification

DC isolation was performed as described previously [8,42]. Briefly, the bone marrow cells were harvested from the femurs and tibias of six- to eight-week-old BALB/c mice on an OVA-free diet and were then cultured in six-well plates (Life Technologies, New York, NY, USA) at density of 3 × 10^5^ cells per well in 2 mL of RPMI-1640 medium (Life Technologies) supplemented with 100 µg/mL streptomycin (Invitrogen, Grand Island, NY, USA), 2 mM l-glutamine (Sigma), 50 mM 2-mercaptoethanol (Sigma) and 10% fetal bovine serum (Life Technologies). DCs, inducing the isolated DCs, were induced by adding 20 ng/mL recombinant mouse granulocyte-macrophage colony-stimulating factor (rmGM-CSF) (R&D Systems, Minneapolis, MN, USA) and 10 ng/mL recombinant murine interleukin 4 (rmIL-4) (R&D Systems) to the culture medium. The medium was refreshed every two days. Non-adherent cells were harvested after seven days. For the rAdVs-mediated genetic modification of DCs, 7-day cultured cells with typical DC clusters were collected and infected with rAdV-CTLA4Ig and rAdV-CCR7 or rAdV-GFP at the optimized multiplicity of infection (MOI) of 100 for 2 h in serum-free medium. Next, these cells were incubated in complete medium containing rmGM-CSF and rmIL-4 for two days. The DCs cultured for seven days without rAdV modification were the control. To generate DCs that presented OVA, all cells were cultured in the presence of 50 µg/mL whole OVA protein (Grade V; Sigma) for two days. The cells were then collected for flow cytometric analysis or for use in subsequent experiments.

### 4.4. Fluorescence-Activated Cell Sorting (FACS) Analysis

CTLA4Ig, CCR7, CD11c, MHC-II and the co-stimulatory molecules CD86 and CD80 expressed on the DC surface were analyzed by FACS (BD Biosciences, San Jose, CA, USA). The cells were stained using the following primary antibodies (mAbs; eBioscience, San Diego, CA, USA): Mouse CCR7 MAb, Human CTLA-4 Biotinylated Affinity Purified PAb, phycoerythrin (PE)-Cy5 conjugated anti-CD11c, PE-conjugated anti-MHC-II, PE-conjugated anti-CD80 and PE-conjugated anti-CD86. After washing with phosphate-buffered saline (PBS), the cells were analyzed using a FACS Calibur flow cytometer (BD FACSAria™ III, BD Biosciences, San Jose, CA, USA). A minimum of 10^4^ events within the gated live population were collected per sample. The data were analyzed with the Cell Quest Pro analysis software (BD Biosciences, San Jose, CA, USA) by gating on the live cell populations. Appropriate isotype-matched antibody controls were used from the respective manufacturers. The infection ratio of the rAdVs infected DCs was verified by GFP expression.

### 4.5. CTLA4Ig and CCR7 Expression Analysis

CTLA4Ig and CCR7 expression in the DCs surface were measured by FACS without 1% TritonX-100 as described above. CTLA4Ig and CCR7 expression in cytoplasm were studied by using immunocellular chemistry after 1% TritonX-100 treatment for 5 min. The Mouse CCR7 MAb (goat) and Human CTLA-4 Biotinylated Affinity Purified PAb (goat) (mAbs; eBioscience, San Diego, CA, USA) were used as primary antibodies, FITC-labeled rabbit anti-goat antibody (mAbs; eBioscience, San Diego, CA, USA) and anti-Mouse Alexa Fluor^®^ 555 (Abcam, Cambridge, MA, USA) were used as second antibodies. CTLA4Ig and CCR7 expression in cytoplasm were verified using a Nikon fluorescent microscope (Nikon, Tokyo, Japan). The level of CTLA4Ig protein in the supernatants of cultured DCs was assayed by enzyme-linked immunosorbent assay (ELISA) (Bender, Vienna, Austria).

### 4.6. Allergen Sensitization, Challenge and Treatment

As reported previously [8], BALB/c mice were divided into the following four groups: healthy control mice group, OVA-sensitized/challenged asthma mice group, OVA-sensitized/challenged mice treated with rAdV-CTLA4Ig- and rAdV-CCR7-infected DCs, and OVA-sensitized/challenged mice treated with rAdV-GFP infected DCs. Each group contained eight mice. For the allergen-induced murine model of asthma, allergen sensitization and inhalational antigen challenge were performed as described previously [8]. Briefly, on days 0 and 14, the mice were sensitized by intraperitoneal and thigh subcutaneous injection of 200 µL of a solution containing 100 µg of OVA mixed with aluminum hydroxide (0.5 mg/mL; Sigma). On days 22–28, the sensitized mice were placed into a 14 × 11 × 11 cm^3^ plastic chamber and were exposed to aerosolized PBS containing 1% OVA (weight/volume) or PBS for 30 min. Healthy control mice received injections of PBS and PBS aerosols in similar to the OVA-treated mice. For mice treated with DCs, prior to the first inhalational antigen challenge of 1 × 10^6^ DCs, rAdV-infected DCs were administered intravenously.

### 4.7. Cytokine Measurement and Cell Subset Analysis

Blood was collected by retro-orbital bleeding and the serum was isolated and stored at −80 °C. The BALF was collected by lavaging the lungs three times with 0.5 mL of PBS, and the cell suspensions were then centrifuged at 1500 rpm for 5 min. After centrifugation, supernatants were collected and stored at −80 °C for cytokine analysis. The BALF cells were resuspended in PBS, and the total leucocyte counts were obtained using a hemocytometer. Differential counts were determined by cytocentrifugation of 30 µL aliquots of BALF cells at 1500 rpm for 3 min onto slides. Next, the slides were stained with Wright–Giemsa (Biovisualab, Shanghai, China) and counted in a blinded fashion. A minimum of 200 cells was counted per sample under light microscopy. The cytokine levels in the BALF and serum were determined using a commercially available ELISA kit according to the manufacturer’s instructions. ELISA kits for IL-4 and interferon (IFN)-γ were purchased from ShiZhengBo (Beijing, China).

### 4.8. Histopathology

Bulging and bloodshot inflammatory cell infiltration and bronchial tube pulmonary alveolus structural changes were verified by histopathology. Hematoxylin and eosin (H&E, Biovisualab, Shanghai, China) staining were performed. The mouse lungs were removed and inflated with 4% paraformaldehyde. The issues were then embedded in paraffin and cut into 5-µm-thick sections. The sections were stained using a standard staining H&E protocol.

### 4.9. CCR7 Guided DC Homing Analysis

To determine whether CCR7 guides DC migration, the sections of mouse lung, kidney and small intestine were used for DC distribution analysis. Because all rAdVs harbor the GFP gene, the GFP expression of rAdV-infected DCs was confirmed in *in vitro* experiments. After transplantation of the DCs infected with rAdV, the homing of rAdV-infected DCs was verified using a Nikon fluorescent microscope (Nikon, Tokyo, Japan), the mean fluorescence intensities were measured and calculated by the fluorescent microscope and carried software.

### 4.10. Statistical Analysis

The results are expressed as the mean ± standard deviation (SD). An analysis of variance (ANOVA) was used to determine the difference between all groups. Pairs of groups were compared using Student’s *t*-test. The results were considered statistically significant for *p*-values <0.05.

## 5. Conclusions

CTLA4Ig-modified DCs exhibited a therapeutic effect on asthma, and CCR7 may guide DC homing. The combination of these two molecules may be a model for precision-guided immunotherapy.
